# On the Construction of Hospitals for the Insane
*These and the subjoined suggestions with reference to the "organization of Hospitals for the Insane," are made by the "Association of Medical Superintendents of American Institutions for the Insane."


**Published:** 1854-04-01

**Authors:** 


### ON THE CONSTRUCTION OF HOSPITALS FOR THE INSANE.*

I. Every hospital for the insane should be in the country, not within less than two miles of a large town, and easily accessible at all seasons.

II. No hospital for the insane, however limited its capacity, should have less than fifty acres of land, devoted to gardens and pleasure-grounds for its patients. At least one hundred acres should be possessed by every State hospital, or other institution for two hundred patients, to which number these propositions apply, unless otherwise mentioned.

III. Means should be provided to raise ten thousand gallons of water, daily, to reservoirs that will supply the highest parts of the building.

IV. No hospital for the insane should be built, without the plan having been first submitted to some physician or physicians, who have had charge of a similar establishment, or are practically acquainted with all the details of their arrangements, and received his or their full approbation.

* These and the subjoined suggestions with reference to the "organization of Hospitals for the Insane," are made by the "Association of Medical Superintendents of American Institutions for the Insane."

V. The highest number that can with propriety be treated in one building is two hundred and fifty, while two hundred is a preferable maximum.

VI. All such buildings should be constructed of stone or brick, have slate or metallic roofs, and, as far as possible, be made secure from accidents by fire.

VII. Every hospital, having provision for two hundred or more patients, should have in it at least eight distinct wards for each sex, making sixteen classes in the entire establishment.

VIII. Each ward should have in it a parlour, a corridor, single lodging-rooms for patients, an associated dormitory, communicating with a chamber for two attendants; a clothes-room, a bath-room, a water-closet, a dining-room, a dumb waiter, and a speaking-tube leading to the kitchen, or other central part of the building.

IX. No apartments should ever be provided for the confinement of patients, or as their lodging-rooms, that are not entirely above ground.

X. No class of rooms should ever be constructed without some kind of window in each, communicating directly with the external atmosphere.

XI. No chamber for the use of a single patient should ever be less than eight by ten feet, nor should the ceiling of any story occupied by patients be less than twelve feet in height.

XII. The floors of patients' apartments should always be of wood.

XIII. The stairways should always be of iron, stone, or other indestructible material, ample in size and number, and easy of ascent, to afford convenient egress in case of accident from fire.

XIV. A large hospital should consist of a main central building with wings.

XV. The main central building should contain the offices, receiving-rooms for company, and apartments entirely private, for the superintending physician and his family, in case that officer resides in the hospital building.

XVI. The wings should be so arranged that, if rooms are placed on both sides of a corridor, the corridors should be furnished at both ends with moveable glazed sashes, for the free admission of both light and air.

XVII. The lighting should be by gas, on account of its convenience, cleanliness, safety, and economy.

XVIII. The apartments for washing clothing, &c., should be detached from the hospital building.

XIX. The drainage should be under ground, and all the inlets to the sewers should be properly secured, to prevent offensive emanations.

XX. All hospitals should be warmed by passing an abundance of pure, fresh air from the external atmosphere, over pipes or plates, containing steam under low pressure, or hot water, the temperature of which at the boiler does not exceed 212° F., and placed in the basement or cellar of the building to be heated.

XXI. A complete system of forced ventilation, in connexion with the heating, is indispensable to give purity to the air of a hospital for the insane; and no expense that is required to effect this object thoroughly can be deemed either misplaced or injudicious.

XXII. The boilers for generating steam for warming the building should be in a detached structure, connected with which may be the engine for pumping water, driving the washing apparatus, and other machinery.

XXIII. All water-closets should, as far as possible, be made of indestructible materials, be simple in their arrangement, and have a strong downward ventilation connected with them.

XXIV. The floors of bath-rooms, water-closets, and basement stories, should, as far as possible, be made of materials that will not absorb moisture.

XXV. The wards for the most excited class should be constructed with rooms on but one side of a corridor, not less than ten feet wide, the external windows of which should be large, and have pleasant views from them.

XXVI. Whenever practicable, the pleasure grounds of a hospital for the insane should be surrounded by a substantial wall, so placed as not to be unpleasantly visible from the building,

---

### ON THE ORGANIZATION OF HOSPITALS FOR THE INSANE.

